# A clinical grade cell-based artificial APT, aAPC/mOKT3, for unbiased expansion of CD3+ T lymphocytes

**DOI:** 10.1186/2051-1426-2-S3-P5

**Published:** 2014-11-06

**Authors:** Valentin Sotov, Toshiki Ochi, Diana Gray, Shlomit Boguslavsky, Vinicius Motta, Naoto Hirano, Marcus Butler

**Affiliations:** 1 Princess Margaret Cancer Centre, Toronto, Canada

## 

Recent clinical results confirm that adoptive cell therapy, whereby expanded antitumor lymphocytes are infused into cancer patients, is a promising new therapy. Standardized methods to reproducibly and efficiently expand high quality antitumor lymphocytes in vitro are needed. We and others have successfully used cell-based artificial APCs (aAPCs) in the clinic as an off-the-shelf, standardized, and renewable reagent to reliably expand antitumor T cells in vitro for adoptive therapy. Recently, we have reported a genetically engineered novel human cell-based aAPC, aAPC/mOKT3, which expresses a membranous form of anti-CD3 mAb (mOKT3) in conjunction with immunostimulatory molecules, CD80 and CD83. Without requiring allogeneic feeder cells, aAPC/mOKT3 induces the robust expansion of both peripheral and tumor-infiltrating T cells, regardless of HLA-restriction, but not Foxp3^+ ^regulatory T cells or NK cells. Expanded T cells predominantly secreted Th1-type cytokines such as interferon-γ and IL-2. Unlike anti-CD3/CD28 mAb-coated beads, aAPC/mOKT3 enabled significantly improved CD8+ T cell expansion in part through IL-21 secreted by cocultured CD4^+ ^T cells. To generate a clinical grade version of this aAPC, the parental cell line K562 was cotransfected with five linearized DNA plasmids encoding the light and heavy chains of mOKT3, CD80, CD83, and a puromycin *N*-acetyl-transferase gene. Simultaneous transfection of the 5 plasmids and subsequent drug selection resulted in aAPC/mOKT3 lines with >20% triple positivity (mOKT3, CD80, and CD83). Using a limiting dilution method, >100 candidate clones were established. Based on high expression of all 3 surface molecules, 41 clones were selected for long-term culture and monitoring for stable, high triple expression. After 3 months of continuous culture, 9 clones demonstrated stable expression and had suitable doubling time (24-40 hours). As seen with research grade aAPC/mOKT3, all clones induced the preferential expansion of CD8^+ ^T cells in the presence of CD4^+ ^T cells. Two clones, which consistently induced superior CD8^+ ^T cell proliferation (>1,000 fold within 4 weeks), are lead candidates for selection to generate a master cell bank of clinical grade aAPC/mOKT3. Further criteria for selection include the ability of clones to expand T cells with a non-exhausted, young phenotype without contaminating immunosuppressive cell populations. Ability of expanded T cells to secrete Th1 cytokines and not immunosuppressive cytokines such as IL-10 or TGF-β will be confirmed. Once these data are obtained, a single clone will be chosen to generate a master cell bank and clinical lots for use in future clinical trials.

